# ROS-Driven STAT1 S-Glutathionylation Sustains IFNγ Signaling and Pro-Inflammatory Microglial Polarization

**DOI:** 10.3390/antiox14121395

**Published:** 2025-11-23

**Authors:** Martina Brattini, Alessandra Carcereri de Prati, Carlotta Passarini, Marta Menegazzi, Alessandra Fiore, Maria Mosaico, Michelle D’Urso, Sofia Mariotto, Elena Butturini

**Affiliations:** Neurosciences, Biomedicine and Movement Sciences, Biological Chemistry Section, University of Verona, Strada le Grazie 8, 37129 Verona, Italy; martina.brattini@univr.it (M.B.); alessandra.carcererideprati@univr.it (A.C.d.P.); carlotta.passarini@univr.it (C.P.); marta.menegazzi@univr.it (M.M.); alessandra.fiore@univr.it (A.F.); maria.mosaico@univr.it (M.M.); michelle.durso@univr.it (M.D.); elena.butturini@univr.it (E.B.)

**Keywords:** IFNγ, STAT1, S-glutathionylation, ROS, neuroinflammation

## Abstract

Oxidative stress is a major driver of neuroinflammation, yet the molecular redox mechanisms that shape microglial activation remain incompletely defined. Among reversible redox modifications, protein S-glutathionylation has emerged as a key regulator of signaling cascades under conditions of elevated Reactive Oxygen Species (ROS). While IFNγ is known to activate STAT1 and promote a pro-inflammatory microglial phenotype, the contribution of oxidative stress to this process is poorly understood. Here, we investigated the interplay between ROS and STAT1 signaling in IFNγ-stimulated microglial cells. We demonstrate that ROS not only enhance STAT1 phosphorylation but also promote its S-glutathionylation, a modification that sustains STAT1 transcriptional activity. This dual regulation leads to prolonged expression of pro-inflammatory mediators, including iNOS, COX2, TNFα, and IL-6. Importantly, STAT1-deficient cells fail to mount these responses, confirming STAT1 as a central redox-sensitive hub in microglial polarization. Our findings identify S-glutathionylation as a molecular switch that links oxidative stress to persistent STAT1 activation and M1 polarization. These results suggest that targeting STAT1 redox regulation could help control microglial overactivation and may offer new opportunities for therapeutic intervention in neuroinflammatory and neurodegenerative diseases.

## 1. Introduction

Microglia, the resident immune cells of the central nervous system, play a crucial role in modulating inflammatory responses. Their activation is a defining feature of neuroinflammation, which is increasingly recognized as a key contributor to the progression of various neurological disorders [1,2]. A range of biological and environmental stimuli such as lipopolysaccharide (LPS) and IFNγ can trigger the pro-inflammatory M1 microglial phenotype, characterized by increased expression of inflammatory proteins (e.g., iNOS, COX2) and surface markers (e.g., CD68, CD86), as well as a morphological shift from a resting ramified state to an amoeboid, phagocytic form. This activation leads to the release of inflammatory cytokines, including IL-23, IL-2, IL-1β, IL-6 and TNFα, along with neurotoxic mediators such as Reactive Oxygen and Nitrogen Species (ROS/RNS) [3,4,5,6]. While initially involved in clearing cellular debris and promoting tissue repair, excessive microglial activation can exacerbate inflammation contributing to neuronal death as well as blood–brain barrier dysfunction.

The classical M1 activation pathway is regulated by several signaling cascades, including nuclear factor kappa-light-chain-enhancer of activated B cells (NF-κB) and signal transducer and activator of transcription 1 (STAT1) [3,7,8,9]. STAT1 is crucial for inflammation control and cell fate regulation, such as apoptosis and proliferation. Activation of STAT1 occurs via phosphorylation at tyrosine 701 (Tyr701) by Janus Kinases (JAKs) following cytokine or growth factor binding to their respective receptors [10]. Once phosphorylated, STAT1 homodimerizes and translocates to the nucleus, where it regulates the expression of target genes such as iNOS, COX2, and caspases (e.g., caspase 2, 3, and 7) [11,12,13]. The STAT1 signaling pathway is negative regulated by various proteins including protein tyrosine phosphatases (PTPs), suppressor-of-cytokine-signaling (SOCS) proteins, and protein inhibitors of activated STATs (PIAS), whose main function is to switch off the signal and prevent excessive or prolonged activation. While proper regulation of STAT1 signaling ensures controlled inflammatory responses, its dysregulation can lead to hyperactivation, contributing to the pathology of neuroinflammatory diseases [14].

In this context, our previous studies identified a link between aberrant STAT1 activation and the pro-inflammatory M1 microglial phenotype. Specifically, oxidative stress in BV2 microglia cells induces both STAT1 phosphorylation and S-glutathionylation, driving its aberrant activation and triggering M1 polarization [7,13,15]. These findings suggest that S-glutathionylation may represent a key mechanism whereby ROS regulate inflammation in neurodegenerative disorders.

Herein, we provide evidence that microglia cells, upon IFNγ stimulation, generate ROS at the initial phases of activation. This early ROS production acts as a key intracellular signal that influences downstream inflammatory pathways and modulates microglial functional states. The increase in ROS levels promotes S-glutathionylation of STAT1 that enhances its activation and sustains persistent M1 microglia polarization. To confirm the central role of STAT1 in this process, we employed STAT1 knockdown in BV2 cells previously generated in our laboratory [7]. In these STAT1-deficient cells, IFNγ exposure for 18 and 24 h failed to induce the expression of M1-associated transcriptional markers or phenotypic features. These findings establish STAT1 as a redox-sensitive molecular hub that integrates ROS signaling with the transcriptional reprogramming required for the establishment and maintenance of the M1 microglial phenotype. Notably, our data support a model in which IFNγ promotes M1 polarization through an early ROS burst that induces STAT1 hyperactivation via S-glutathionylation. This redox-dependent mechanism may represent an initiating event in the pathophysiology of neuroinflammatory disorders, where sustained microglial activation contributes to disease progression.

## 2. Materials and Methods

### 2.1. Chemicals

All chemicals used throughout the present study were of the highest analytical grade and purchased from Merck, Milan, IT, unless otherwise specified.

### 2.2. Cell Culture

All experiments were conducted using murine cultured microglial BV2 cells (ICLC, Interlab Cell Line Collection, Genova, Italy). These cells serve as a reliable model for primary microglia and brain-resident microglia, as they exhibit a similar ion channel expression profile and the ability to generate Reactive Oxygen Species (ROS) [16,17]. BV2 cells were cultured in Dulbecco’s modified Eagle media (DMEM, Thermo Fisher Scientific, Monza, Italy) supplemented with 10% FBS, 100 IU/mL penicillin, 100 µg/mL streptomycin and 40 µg/mL gentamycin in a 5% CO_2_ humidified atmosphere at 37 °C. Murine cultured microglial STAT1 knockdown in BV2 cell line was generated by lentiviral transduction as previously described in [7]. STAT1 knockdown in BV2 cells were cultured in DMEM supplemented with 5% FBS, 100 IU/mL penicillin, 100 µg/mL streptomycin, 40 µg/mL gentamycin and 4 µg/mL puromycin in a 5% CO_2_ humidified atmosphere at 37 °C.

### 2.3. Measurement of Intracellular Reactive Oxygen Species

ROS production in BV2 cells was assessed with the cell-permeable probe 2′,7′-dichlorodihydrofluorescein diacetate (H_2_DCFDA; Thermo Fisher Scientific). First, BV2 cells were seeded in 96-well plate, left to adhere overnight until 90% confluent and then loaded with 10 μM H_2_DCFDA. After incubation for 1 h, the cells were treated with 20 ng/mL IFNγ for the indicated time. Fluorescence intensity was measured with a multimode plate reader (Ex_485nm_ and Em_535nm_) (Infinite N Nano, Tecan Trading). Fluorescence intensity was normalized against control wells for statistical analysis.

### 2.4. Glutathione Content Quantification

The intracellular reduced glutathione (GSH) concentration was measured by endpoint spectrophotometric titration on a Jasco V/550 spectrophotometer (JASCO, Cremella, Italy) using 5,5′-dithiobis (2-nitrobenzoic acid) (DTNB, Ellman’s reagent) [18,19]. Briefly, treated and untreated cells were lysed by freezing and thawing in 100 mM sodium phosphate buffer, pH 7.5, containing 5 mM EDTA (KPE buffer), and after centrifugation at 16,000 rpm for 10 min, total protein concentration was determined by using the Bradford method [20]. The supernatants were deproteinized with 5% trichloroacetic acid. For GSH measurement, acidified clear supernatants were neutralized and buffered at pH 7.4 with 200 mM K_2_HPO_4_, pH 7.5. The reaction was then started by the addition of 60 μM DTNB, and the increase in absorbance at 412 nm was measured until no variation in absorbance was evident. The amount of total GSH was determined by comparison with the GSH standard curve.

### 2.5. Western Blot Analysis

Cells were homogenized at 4 °C in 20 mm HEPES, pH 7.4, containing 420 mm NaCl, 1 mm EDTA, 1 mm EGTA, 1% Igepal, 20% glycerol, protease cocktail inhibitors, and phosphatase cocktail inhibitors. Protein concentration was estimated by Coomassie Protein assay reagent (Thermo Fisher Scientific), with reference to bovine serum albumin (BSA) standards. Protein extracts (50 μg total proteins/lane) were resolved by SDS-polyacrylamide gel electrophoresis and transferred onto a polyvinylidene difluoride (PVDF) membrane (Immobilon P, Millipore, Bedford, MA, USA). Membranes were blocked with 5% BSA or 5% fat dry milk in Tris-buffered saline with 0.1% Tween 20 (TBST) at room temperature for 1 h and then incubated with primary antibodies specific for anti-phospho-Tyr701 STAT1 (sc-136229 Santa Cruz Biotechnology, Santa Cruz, CA, USA), anti-STAT1 (sc-346, Santa Cruz Biotechnology, Santa Cruz, CA, USA), anti-Actin (Santa Cruz Biotechnology, Santa Cruz, CA, USA), anti-Tubulin (Oncogene, CP06-100UG), and anti-iNOS (ab-15323, Abcam, Cambridge, UK). After washing with TBST, the membranes were hybridized with anti-rabbit or anti-mouse IgG peroxidase-conjugated secondary antibody (S7074, S7076, Cell Signalling Technology, Danvers, MA, USA)) and developed by Western Chemiluminescent HRP Substrate (WBKLS0500, Millipore) using the ChemiDoc XRS Imaging System (BioRad, Hercules, CA, USA). Blotted proteins were quantified using ImageLab 6.1.0 (BioRad).

### 2.6. Enzyme-Linked Immunosorbent Assay (ELISA)

To quantify the release of pro-inflammatory cytokines, BV2 cells were treated with 20 ng/mL IFNγ for 18 h. At the end of the treatment, culture supernatants were collected and centrifuged at 1000× *g* for 10 min to remove cell debris. The concentrations of IL-6 and TNFα were measured using commercially available ELISA kits specific for mouse cytokines, following the manufacturer’s instructions (88-7064-88, 88-7324-88, Thermo Fisher Scientific). Absorbance was read at 450 nm using a microplate reader, and cytokine concentrations were calculated based on standard curves included in each assay.

### 2.7. Immunoprecipitation and Identification of Glutathionylated Proteins

Cells were lysed in RIPA buffer (20 mm Tris/HCl pH 8.0, 150 mm NaCl, 1% Igepal, 1 mm EDTA, 10% glycerol, 100 mm NaF, and 1 mm Na_3_VO_4_) supplemented with protease inhibitor cocktail for 30 min on ice. Equal amounts of proteins from the clarified cell lysates were incubated overnight at 4 °C with rotation in the presence of anti-STAT1 antibody. The immune complexes were collected by addition of protein A Sepharose (GE Healthcare, Pittsburg, PA, USA), washed extensively, eluted in a nonreducing sample buffer (62.5 mm Tris/HCl pH 6.8, 10% glycerol, 5% SDS, and 0.05% bromophenol blue), separated by SDS-polyacrylamide gel electrophoresis and transferred onto a PVDF membrane, and analyzed as previously described [15]. Briefly, membranes were probed with primary monoclonal antibody against GSH (ViroGen 101-A-100) and, after washing, blots were incubated with anti-mouse IgG peroxidase-conjugated antibody (Cell Signaling Technology, Danvers, MA, USA). Protein-antibody reactions were detected with Western Chemiluminescent HRP Substrate (WBKLS0500, Millipore). The S-glutathionylated proteins on membranes were detected using the ChemiDoc XRS Imaging System (BioRad). After stripping, membranes were re-hybridized with rabbit anti-STAT1antibody (Santa Cruz Biotechnology).

### 2.8. Immunofluorescence and Confocal Analysis

BV2 microglial cells onto the coated glass were fixed with 4% paraformaldehyde (PFA) for 10 min and washed three times with PBS. BV2 cells were permeabilized with 0.1% Triton X-100 in PBS for 5 min and blocked with 5% bovine serum albumin (BSA) for 1 h. And then, samples were incubated with primary antibodies anti-pTyr701-STAT1 (Santa Cruz Biotechnology) overnight at 4 °C. After incubation, cells were washed three times for 3 min with PBS, incubated with secondary antibody (Alexa Fluor^®^ 594 anti-mouse) for 1 h at room temperature, and counterstained with 4′,6-diamidino-2-phenylindole (DAPI, 1:1000, Thermo Fisher Scientific) for 15 min at room temperature. Cell images were captured using an EVIDENT FV4000 Inverted confocal microscope at 60× magnification.

### 2.9. RT-qPCR Analysis

Total cellular RNA was extracted using the Pure Link RNA isolation kit (ID:12183018A, Thermo Fisher Scientific) quantified at 260/280 nm and tested by 1% agarose gel electrophoresis to check the integrity of the samples. Aliquots corresponding to 1 µg of total RNA were reverse transcribed by using the SuperScriptVilo cDNA synthesis kit (ID: 11754-(50), Thermo Fisher Scientific) following the manufacturer’s protocol. The cDNAs (corresponding to 25 ng of the original RNA) were subjected to real-time PCR with the QuantiTect SYBR Green PCR Kit (ID: 204143, Qiagen, Valencia, CA, USA) following the manufacturer’s instructions. The mRNA levels of STAT1-regulated genes, including COX2 and iNOS, were analyzed by quantitative real-time PCR. GAPDH was used as the internal control.

### 2.10. Statistical Analysis

The results are expressed as the mean ± SD of at least four independent experiments. When only two groups were compared, Student’s *t*-test was used to determine significance, and *p* < 0.05 was considered statistically significant.

## 3. Results

### 3.1. IFNγ-Induced M1 Phenotype Activation in BV2 Cells Through Oxidative Stress

**Figure 1:**
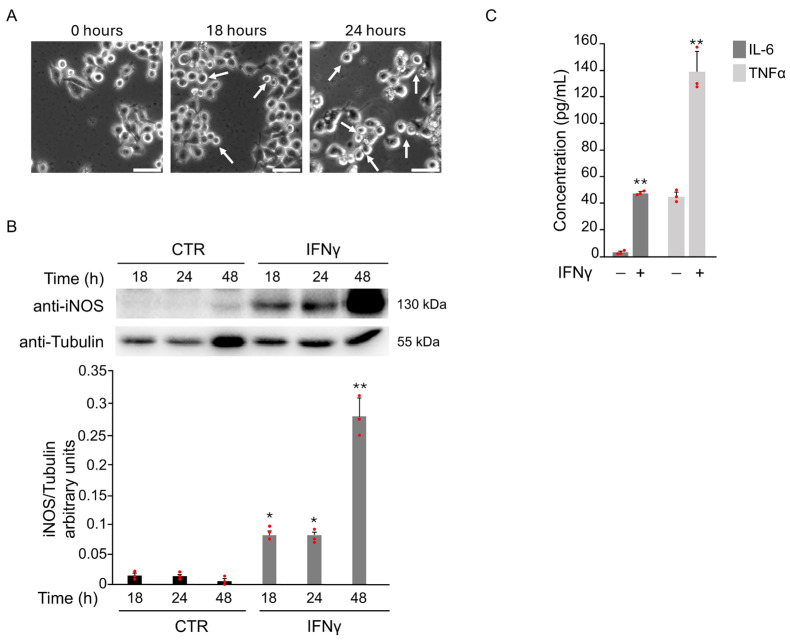
IFNγ treatment induces pro-inflammatory M1 phenotype in BV2 cells. BV2 cells were exposed to 20 ng/mL of IFNγ for the indicated time points, and different parameters were evaluated to confirm the induction of M1 microglia phenotype. (**A**) Phase-contrast microscopy images of BV2 cells cultured in the presence of IFNγ showed a morphological change from a branched to an ameboid shape (white arrows) at 18 and 24 h after the treatment (20×). Scale bars: 50 μm. (**B**) Total protein extracts were analyzed by Western Blot with anti-iNOS and anti-Actin antibody. As confirmed by densitometric analysis, iNOS protein levels increased from 18 h until 48 h after stimulation with IFNγ with respect to untreated BV2 cells (CTR). (**C**) The expression of the pro-inflammatory cytokines IL-6 and TNFα in the supernatant of BV2 cells was evaluated by ELISA after 18 h of IFNγ stimulation. Data are expressed as mean ± SD (n = 3). * *p* < 0.05, ** *p* < 0.01 vs. CTR. Single data points are shown as red dots.

Microglial activation plays a key role in neurodegenerative diseases. Oxidative stress, triggered by pro-inflammatory cytokines or brain ischemia, contributes to this process and promotes the shift toward a pro-inflammatory M1 phenotype. As reported in previous studies [7], BV2 cells showed typical M1 morphology at 18 and 24 h after treatment with 20 ng/mL IFNγ (Figure 1A).

To confirm M1 activation, iNOS expression was assessed. A marked increase in iNOS levels was detected starting from 18 h after IFNγ exposure (Figure 1B). The levels of IL-6 and TNFα in the supernatants of BV2 cells after IFNγ treatment for 18 h further support this. ELISA results revealed a significant increase in both cytokines compared to untreated controls (Figure 1C).

**Figure 2:**
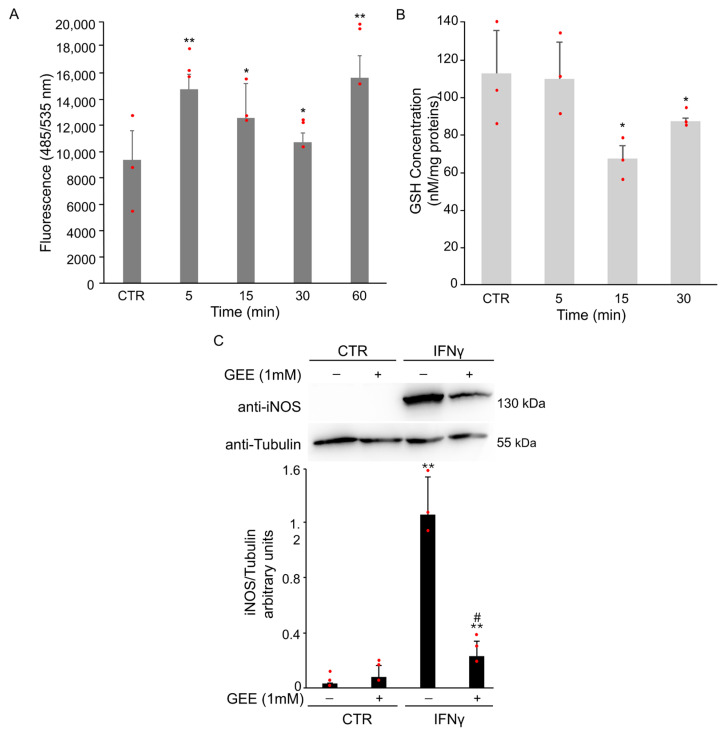
IFNγ treatment induces oxidative stress and M1 polarization in BV2 cells. (**A**) BV2 cells were pre-treated with the H_2_DCFDA probe and then exposed to 20 ng/mL of IFNγ for the indicated time. Intracellular ROS production was evaluated by measuring the fluorescent intensity (Ex485 nm and Em535 nm). Data are expressed as mean ± SD (n = 4). * *p* < 0.05, ** *p* < 0.01 vs. untreated BV2 cells (CTR). (**B**) Intracellular GSH levels were spectrophotometrically analyzed by DTNB. Data are expressed as mean ± SD (n = 3). * *p* < 0.05 vs. untreated BV2 cells (CTR). (**C**) BV2 cells were pre-treated overnight with 1 mM GEE and then exposed to 20 ng/mL of IFNγ for 18 h. Total protein extracts were analyzed by Western Blot with anti-iNOS antibody. The same blot was incubated with anti-Tubulin antibody to check the amount of loaded proteins. As shown by densitometric analysis, pre-treatment with GEE partially reduced iNOS activation compared to IFNγ treatment alone. Images are representative of three independent experiments. ** *p* < 0.01 vs. untreated BV2 cells (CTR). # *p*< 0.05 vs. IFNγ-treated cells. Single data points are shown as red dots.

To explore the role of oxidative stress in this process, intracellular ROS levels were monitored using cell-permeable ROS-specific fluorescent probe H_2_DCFDA. ROS accumulation was evident as early as 5 min after IFNγ treatment (Figure 2A). Moreover, intracellular GSH levels were evaluated at selected time points using a spectrophotometric assay. A rapid and significant decrease in GSH was observed at 15 min after IFNγ stimulation (Figure 2B), in line with the increase in ROS levels.

To determine whether oxidative stress contributes to M1 polarization, BV2 cells were pretreated overnight with 1 mM Glutathione Ethyl Ester (GEE), a cell-permeable antioxidant, before treatment for 18 h with IFNγ. Pretreatment with GEE resulted in a partial reduction in iNOS expression compared to IFNγ treatment alone (Figure 2C).

Together, these findings indicate that IFNγ promotes M1 microglial activation, at least in part, through the induction of oxidative stress. To better understand the molecular mechanisms underlying this redox-dependent activation, we next focused on STAT1 signaling, a key pathway known to mediate the pro-inflammatory effects of IFNγ.

#### 3.1.1. IFNγ Triggers Phosphorylation of STAT1 in BV2 Cells

**Figure 3:**
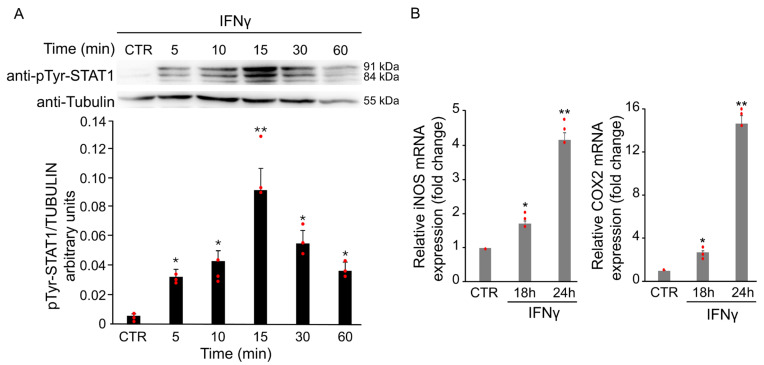
IFNγ treatment induces STAT1 activation in BV2 cells. BV2 cells were exposed to 20 ng/mL of IFNγ at different time points and activation of STAT1 was evaluated through different experiments. (**A**) Total protein extracts of IFNγ-treated BV2 cells at different time points were analyzed by Western Blot with anti-phospho-Tyr701 STAT1 antibody, and after stripping, the same blot was analyzed with anti-STAT1 antibody. Densitometric analysis revealed a STAT1 phosphorylation upon IFNγ treatment, peaking at 15 min compared with untreated BV2 cells (CTR). (**B**) RT-qPCR of iNOS and COX2, two STAT1-dependent genes, revealed an increase in their mRNA levels after 18 and 24 h of IFNγ treatment in BV2 cells. Data are expressed as mean ± SD (n = 3). * *p* < 0.05, ** *p* < 0.01 vs. untreated cells (CTR). Single data points are shown as red dots.

Cytokines and oxidative stress are known to induce aberrant STAT1 activation in several neurological disorders, contributing to inflammation and neuronal damage. Phosphorylation at tyrosine 701 is a key step for STAT1 activation, dimer formation, and nuclear translocation. To assess the effect of IFNγ on STAT1 signaling, total protein extracts from BV2 cells were analyzed by Western Blot using a specific antibody against Tyr701-phosphorylated STAT1. As shown in Figure 3A, phosphorylation of STAT1 increased after treatment with 20 ng/mL IFNγ, peaking at 15 min. The increase was already detectable at 5 min, persisted up to 1 h, followed by a gradual decrease. No change in total STAT1 protein levels was observed at any time point analyzed (Figure 3A).

To further confirm the activation of STAT1 upon IFNγ treatment, mRNA levels of iNOS and COX2 were evaluated by RT-qPCR. As shown in Figure 3B, the treatment with 20 ng/mL of IFNγ for 18 and 24 h induced a time-dependent increase in the expression of both genes.

Since these results demonstrate that IFNγ activates STAT1 signaling, we next investigated whether oxidative stress plays a role in modulating STAT1 phosphorylation.

#### 3.1.2. IFNγ Alters the Intracellular Redox State and Triggers STAT1 Phosphorylation in BV2 Cells

**Figure 4:**
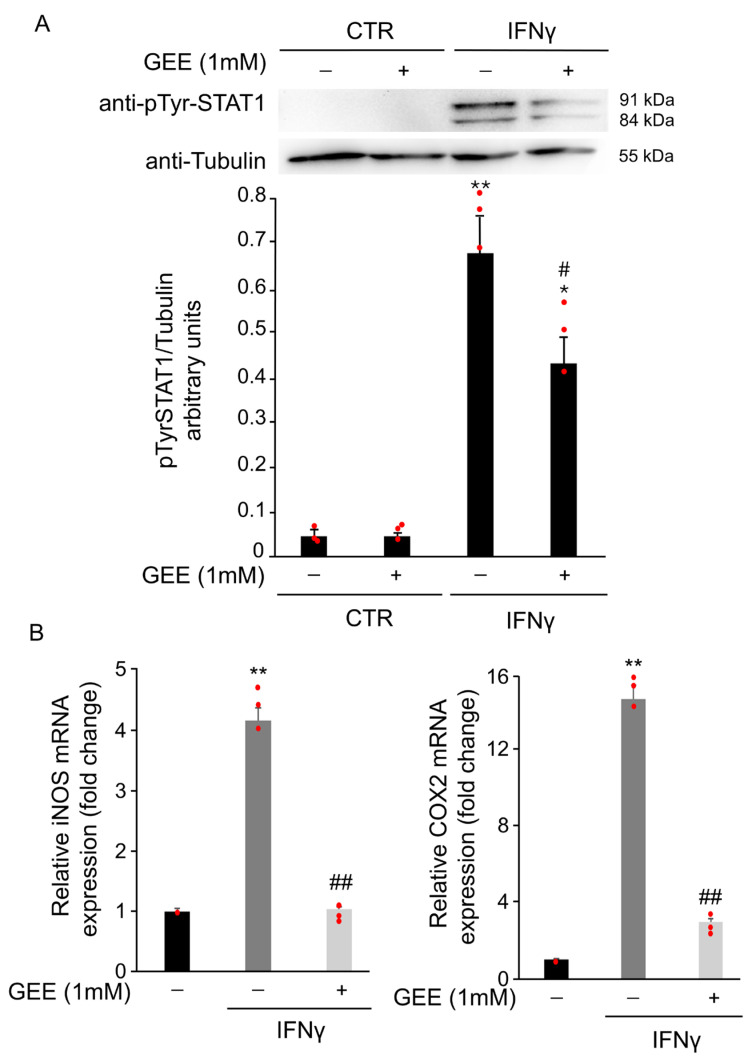
GEE pre-treatment counteracts IFNγ-dependent STAT1 activation in BV2 cells. (**A**) Total protein extracts from BV2 cells pre-treated overnight with GEE and subsequently exposed to 20 ng/mL IFNγ for 18 h were analyzed by Western Blot using a phospho-Tyr701 STAT1 antibody. The same blot was analyzed with anti-Tubulin antibody to check the amount of loaded proteins. Densitometric analysis showed decreased STAT1 phosphorylation in GEE pre-treated cells compared to CTR. * *p* < 0.05, ** *p* < 0.01 vs. untreated BV2 cells (CTR). # *p* < 0.05 vs. IFNγ-treated cells. (**B**) RT-qPCR analysis revealed that iNOS and COX2 mRNA levels were reduced in BV2 cells pre-treated overnight with GEE and then exposed to 20 ng/mL IFNγ for 24 h. Data are expressed as mean ± SD (n = 3). ** *p* < 0.01 vs. CTR, ## *p* < 0.01 vs. IFNγ-treated cells. Single data points are shown as red dots.

To assess whether IFNγ-induced oxidative stress contributes to STAT1 phosphorylation, BV2 cells were pretreated overnight with 1 mM GEE before exposure to IFNγ for 18 h. GEE pretreatment partially reduced phosphorylation at tyrosine 701 compared to IFNγ alone (Figure 4A). Moreover, RT-qPCR results showed that pre-treatment with GEE reduced iNOS and COX2 mRNA levels in BV2 cells compared with IFNγ treatment alone for 24 h, further supporting the reduced STAT1 activity in the presence of GEE (Figure 4B).

**Figure 5:**
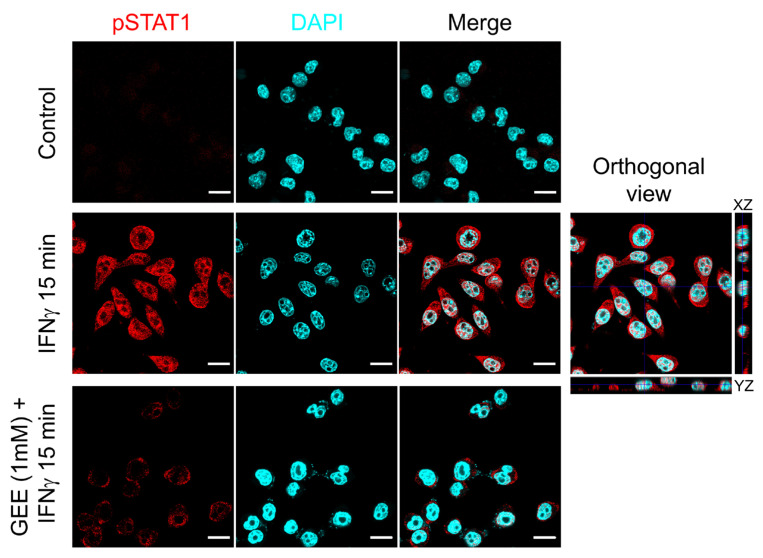
GEE pre-treatment inhibits IFNγ-induced STAT1 phosphorylation and nuclear translocation in BV2 cells. The cells were immunostained with phospho-Tyr701 STAT1 (red) and analyzed by confocal microscopy (lens 60×). Nuclei were stained with DAPI (cyan). The orthogonal sections (ZX and ZY) of merge image reveal that pTYR^701^ STAT1 protein is inside the nuclei. Scale bars indicate 10 μm. Images are representative of three separate experiments.

As a further confirmation of the effect of GEE on STAT1 activation, Figure 5 shows a confocal image where phosphorylated STAT1 is visualized in red. As observed, GEE pretreatment markedly reduces STAT1 phosphorylation and prevents its nuclear translocation induced by IFNγ.

These results confirm that IFNγ induces STAT1 phosphorylation, at least in part, through the generation of oxidative stress. The partial reduction in Tyr701 phosphorylation following antioxidant pretreatment suggests that redox imbalance contributes to STAT1 activation, supporting the idea that oxidative signals cooperate with canonical pathways to regulate STAT1 function in microglia. Based on this redox sensitivity, we then investigated whether oxidative stress could directly influence STAT1 activity through specific redox-dependent post-translational modifications.

#### 3.1.3. IFNγ Alters the Intracellular Redox State and Induces S-Glutathionylation of STAT1 in BV2 Cells

Previous studies showed that changes in the intracellular redox state activate STAT1 signaling through S-glutathionylation at Cys324 and Cys492 [15]. This redox-dependent modification may regulate STAT1 signaling in IFNγ-stimulated microglia.

**Figure 6:**
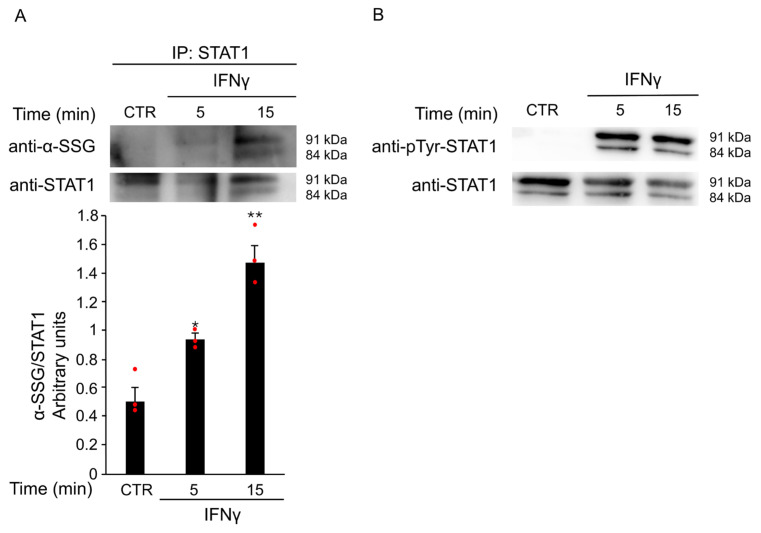
IFNγ treatment induces S-glutathionylation of STAT1. (**A**) Total protein extracts of BV2 cells left untreated (CTR) or treated with 20 ng/mL IFNγ for the indicated times were subjected to immunoprecipitation with anti-STAT1 antibody. Immunoprecipitated STAT1 (IP: STAT1) was analyzed by Western Blot under non-reducing conditions using anti-SSG antibody and, after membrane stripping, re-probed with anti-STAT1 antibody. Densitometric analysis revealed increased STAT1 S-glutathionylation after 5 and 15 min of IFNγ treatment compared to untreated BV2 cells. * *p* < 0.05, ** *p* < 0.01 vs. untreated BV2 cells (CTR). (**B**) Total protein lysates from the same untreated and treated BV2 cells were reserved before pull-down (input) and analyzed by Western Blot with anti-phospho-Tyr701 STAT1 antibody and, after membrane stripping, re-probed with anti-STAT1 antibody. Images are representative of four independent experiments. Single data points are shown as red dots.

To investigate whether ROS production triggered by IFNγ contributes to STAT1 activation via S-glutathionylation, BV2 cells were treated with 20 ng/mL IFNγ for different time points. Total protein extracts were immunoprecipitated with an anti-STAT1 antibody. Western Blot analysis revealed a time-dependent increase in STAT1 S-glutathionylation, with a peak at 15 min after stimulation (Figure 6A). Pretreatment with GEE effectively reversed this post-translational modification (Supplementary Figure S1), further supporting the key role of oxidative stress in STAT1 activation. In addition, as shown in the input panel in Figure 6B, the same samples also showed STAT1 phosphorylation, confirming that both post-translational modifications, S-glutathionylation and phosphorylation, occur simultaneously on the STAT1 transcription factor. Together, these results indicate that IFNγ promotes STAT1 activation through complementary redox-dependent mechanisms, suggesting a tight interplay between oxidative stress and canonical phosphorylation pathways. To explore the functional significance of these findings, we next investigated whether STAT1 is required for IFNγ-induced M1 polarization in microglia.

#### 3.1.4. IFNγ Triggers M1 Phenotype in BV2 Cells Through the Activation of Oxidative Stress and STAT1 Signaling

**Figure 7:**
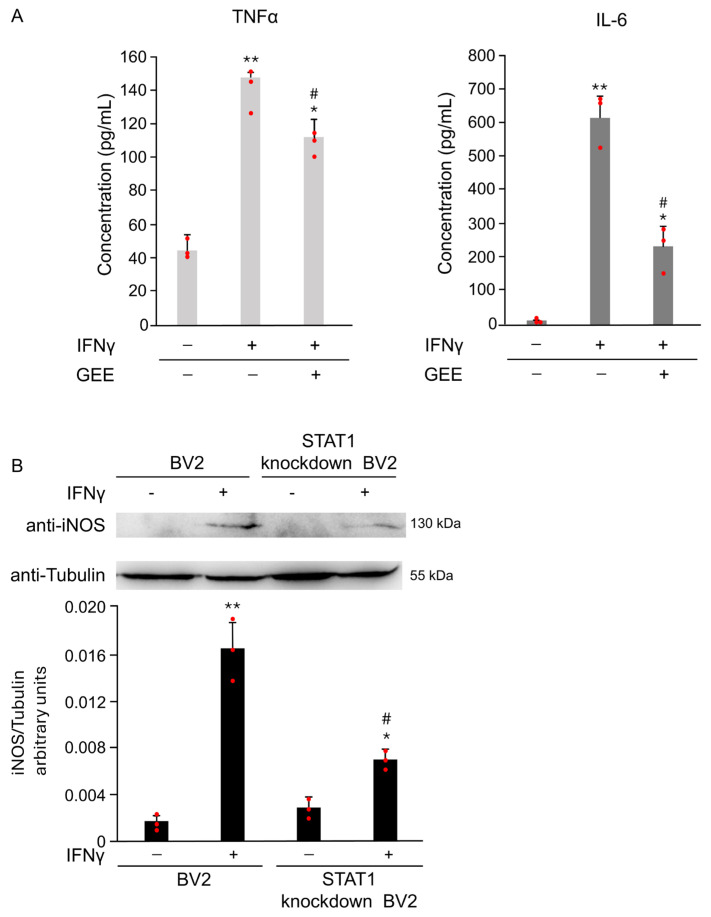
FNγ induces M1 polarization in BV2 cells through oxidative stress and STAT1 activation. (**A**) The expression of the pro-inflammatory cytokines IL-6 and TNFα in the supernatant of BV2 cells was evaluated by ELISA after 18 h of IFNγ stimulation, with or without GEE pretreatment. GEE markedly reduced the IFNγ-induced secretion of both cytokines, confirming the involvement of oxidative stress in M1 polarization. Data are expressed as mean ± SD (n = 3). * *p* < 0.05, ** *p* < 0.01 vs. CTR. # *p* < 0.05 vs. IFNγ. (**B**) Parental and STAT1 knockdown in BV2 cells were treated with 20 ng/mL of IFNγ for 18 h. Total protein extracts were analyzed by Western Blot with anti-iNOS and anti-Actin antibodies. Densitometric analysis revealed reduced iNOS induction in STAT1 knockdown in BV2 cells compared with parental cells upon IFNγ treatment. Parental and STAT1 knockdown in BV2 cells cultured without IFNγ were used as controls (CTR). Images are representative of three independent experiments. Single data points are shown as red dots. * *p* < 0.05, ** *p* < 0.01 vs. CTR. # *p* < 0.05 vs. IFNγ.

Oxidative stress plays a crucial role in IFNγ-induced microglial activation. As shown in Figure 7A, pretreatment with the antioxidant GEE markedly reduced the secretion of the pro-inflammatory cytokines IL-6 and TNFα induced by IFNγ, confirming that oxidative imbalance contributes to M1 polarization. To further understand the signaling mechanisms underlying this effect, we focused on STAT1, a key transcription factor that regulates pro-inflammatory gene expression in microglia.

To directly assess its role in IFNγ-induced M1 activation, we compared the response of parental and STAT1 knockdown in BV2 cells to IFNγ stimulation. As described in [15] and shown in Supplementary Figure S2, STAT1 knockdown in BV2 cells exhibit significantly lower STAT1 expression compared to parental cells. As shown in Figure 7B, STAT1 knockdown in BV2 cells treated with 20 ng/mL IFNγ for 18 h exhibited markedly lower iNOS expression compared to the parental cells exposed to the same conditions. Densitometric analysis of the Western Blot confirmed a significant reduction in iNOS levels in STAT1 knockdown in BV2 (Figure 7B). These results demonstrate that STAT1 plays a central role in driving IFNγ-mediated microglial activation and highlight STAT1 as a critical mediator of the M1 pro-inflammatory phenotype

## 4. Discussion

In the present study, we demonstrate that IFNγ-induced activation of BV2 microglial cells towards the M1 phenotype is critically mediated by oxidative stress and sustained by a redox-dependent mechanism involving STAT1 S-glutathionylation. Specifically, we show that IFNγ stimulation triggers an early burst of ROS production, accompanied by a rapid depletion of intracellular GSH, which in turn promotes both STAT1 phosphorylation and S-glutathionylation. This dual modification enhances STAT1 transcriptional activity and sustains the expression of pro-inflammatory genes, including iNOS and COX2. Importantly, genetic ablation of STAT1 completely abolished M1 polarization, confirming that STAT1 functions as an essential redox-sensitive hub in the IFNγ signaling cascade. These results provide a clear mechanistic link between oxidative stress and the pro-inflammatory activation of microglia, advancing our understanding of how redox imbalance contributes to neuroinflammatory processes.

Our group was the first to identify STAT1 S-glutathionylation in microglia, establishing this reversible oxidative modification as a novel regulatory mechanism of STAT1 function [15]. The current findings extend that work by demonstrating that IFNγ-driven ROS not only trigger STAT1 phosphorylation, the canonical activating event, but also promote its S-glutathionylation, which stabilizes STAT1 activity and prolongs transcriptional responses. In line with our previous study, where we showed that this oxidative modification maintains STAT1 phosphorylation and preserves its functional activity, the present data suggest that S-glutathionylation may play a key role in sustaining STAT1 signaling under neuroinflammatory conditions. Given that STAT1 is frequently overactivated in such contexts [21], the persistence of this redox-dependent modification could contribute to its aberrant activation, thereby amplifying the pro-inflammatory response of microglia. This evidence highlights STAT1 S-glutathionylation as a crucial molecular event that not only regulates microglial activation but may also serve as a potential target to modulate excessive inflammatory responses in the central nervous system. From a broader perspective, this mechanism represents a novel paradigm in redox signaling, showing how oxidative stress can directly influence cytokine-driven pathways and maintain inflammation over time.

The role of oxidative stress in microglial activation has long been recognized. Pro-inflammatory stimuli such as LPS and IFNγ increase ROS production, which contributes to neurotoxicity and persistent inflammation [22,23,24,25]. Our observation that early ROS production is required for full STAT1 activation is consistent with previous evidence that ROS act as upstream mediators of iNOS induction in activated microglia [26,27,28]. Conversely, other reports have suggested that ROS function mainly as downstream by-products of cytokine signaling [29]. The partial reduction in STAT1 phosphorylation and iNOS expression upon antioxidant treatment in our experiments supports the concept that ROS actively modulate STAT1 signaling, rather than serving as passive indicators of oxidative stress. Overall, these results indicate that oxidative stress plays an active and dynamic role in regulating microglial activation, acting as both a trigger and a sustaining factor in the inflammatory response.

While STAT1 is generally considered a pro-inflammatory mediator, its role can vary across different cellular contexts. In some models, STAT1 deficiency has been associated with exaggerated inflammatory responses due to compensatory activation of NF-κB or STAT3 [30,31]. In contrast, we found that STAT1 knockdown in BV2 cells completely failed to acquire an M1 phenotype after IFNγ stimulation, confirming that in microglia STAT1 is indispensable for pro-inflammatory polarization. This highlights the importance of cell type-specific mechanisms in determining STAT1 signaling outcomes. Our results therefore strengthen the concept that microglial STAT1 plays a unique and non-redundant role in orchestrating neuroinflammation, suggesting that selective modulation of its redox state could represent a promising therapeutic approach.

Finally, our results provide mechanistic insight into the persistence of microglial activation in neuroinflammatory conditions. We propose that STAT1 S-glutathionylation may act as a form of “redox memory”, maintaining STAT1 in a hyperactivated state and thereby prolonging the M1 phenotype. Such a mechanism could contribute to the chronic microglial activation observed in Alzheimer’s disease, multiple sclerosis, and Parkinson’s disease, all of which are characterized by heightened oxidative stress [32,33]. By identifying this form of redox memory, our study bridges molecular mechanisms with disease pathophysiology, suggesting that targeting STAT1 S-glutathionylation might help prevent or attenuate chronic neuroinflammation in these disorders.

Taken together, our findings identify STAT1 S-glutathionylation as a recently characterized mechanism contributing to IFNγ-induced microglial polarization. Beyond its mechanistic significance, this discovery offers translational potential, as modulation of STAT1 redox regulation could serve as a novel strategy to counteract oxidative stress-driven inflammation. By linking oxidative stress to sustained STAT1 activation, this work advances the molecular understanding of redox-dependent regulation in neuroinflammation and provides a rationale for targeting STAT1 glutathionylation as a potential therapeutic approach in neurodegenerative disorders.

## Data Availability

The original contributions presented in this study are included in the article/supplementary material. Further inquiries can be directed to the corresponding author.

## Supplementary Materials

The following supporting information can be downloaded at: https://www.mdpi.com/article/10.3390/antiox14121395/s1, Figure S1: GEE pretreatment reduces IFNγ-induced STAT1 S-glutathionylation in BV2 cells; Figure S2: Lentiviral transduction of BV2 cells allows STAT1 silencing

## Abbreviations

The following abbreviations are used in this manuscript:

GEE	Glutathione Ethyl Ester
ROS	Reactive Oxygen Species
GSH	Glutathione
IFNγ	Interferon gamma
STAT1	Signal transducer and activator of transcription 1
