# Supplementary material for: ROS-Driven STAT1 S-Glutathionylation Sustains IFNγ Signaling and Pro-Inflammatory Microglial Polarization
Source: Antioxidants (Basel). 2025 Nov 23;14(12):1395. doi: 10.3390/antiox14121395 (PMC12729941; doi:10.3390/antiox14121395)
Supplement: Supplementary file 1 [file antioxidants-14-01395-s001.zip › antioxidants-3960976-supplementary.pdf]

Supplementary material

ROS-driven STAT1 S-glutathionylation sustains IFNγ signaling and pro-inflammatory microglial polarization

Brattini M. [1], Carcereri de Prati A. [1], Passarini C. [1], Menegazzi M. [1], Fiore A. [1], Mosaico M., D'Urso M., Mariotto S. [1*] and Butturini E.

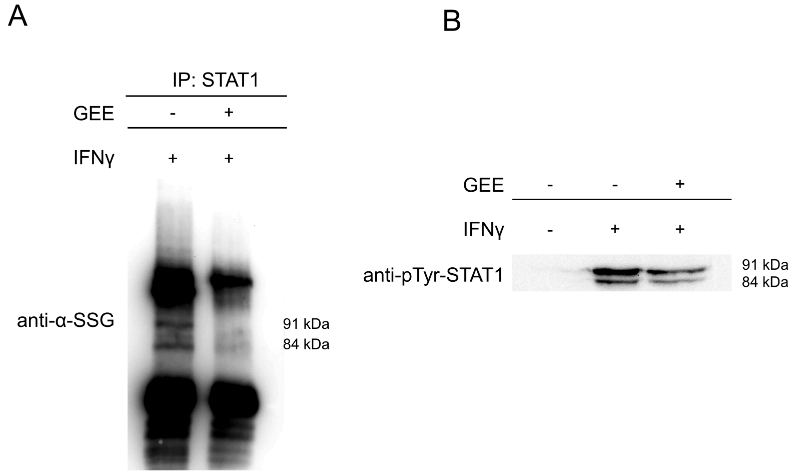

Supplementary 1. GEE pretreatment reduces IFNγ-induced STAT1 S-glutathionylation in BV2 cells. A) Total protein extracts from BV2 cells pretreated or not with 1 mM GEE (overnight) and then stimulated with 20 ng/mL IFNγ for 15 minutes were subjected to immunoprecipitation with anti-STAT1 antibody. Immunoprecipitated STAT1 (IP: STAT1) was analyzed by Western blot under non-reducing conditions using anti-SSG antibody. **B)** Total protein lysates from the same cells were reserved before pull-down (input) and analyzed by Western Blot with anti-phospho-Tyr701 STAT1 antibody.

GEE pretreatment markedly reduced IFNγ-induced STAT1 S-glutathionylation and TYR phosphorylation.

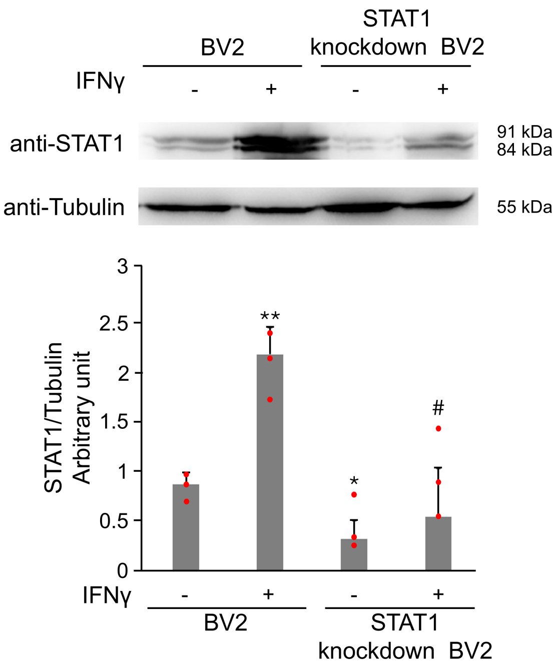

Supplementary 2. Lentiviral transduction of BV2 cells allows STAT1 silencing. Parental and STAT1 knockdown in BV2 cells were exposed to 20 ng/mL of IFNγ for 18 hours, and total protein extracts were analysed by Western Blot with anti-STAT1 and with anti-actin antibodies. Densitometric analysis revealed decreasing levels of STAT1 in both untreated and IFNγ-treated STAT1 knockdown in BV2 cells with respect to parental cells.
